# Phosphatidylinositol 5-Phosphate Links Dehydration Stress to the Activity of *ARABIDOPSIS* TRITHORAX-LIKE Factor ATX1

**DOI:** 10.1371/journal.pone.0013396

**Published:** 2010-10-13

**Authors:** Ivan Ndamukong, David R. Jones, Hanna Lapko, Nullin Divecha, Zoya Avramova

**Affiliations:** 1 School of Biological Sciences, University of Nebraska at Lincoln, Lincoln, Nebraska, United States of America; 2 Inositide Laboratory, Paterson Institute for Cancer Research, The University of Manchester, Manchester, United Kingdom; Iwate University, Japan

## Abstract

**Background:**

Changes in gene expression enable organisms to respond to environmental stress. Levels of cellular lipid second messengers, such as the phosphoinositide PtdIns5*P*, change in response to a variety of stresses and can modulate the localization, conformation and activity of a number of intracellular proteins. The plant trithorax factor (ATX1) tri-methylates the lysine 4 residue of histone H3 (H3K4me3) at gene coding sequences, which positively correlates with gene transcription. Microarray analysis has identified a target gene (*WRKY70*) that is regulated by both ATX1 and by the exogenous addition of PtdIns5*P* in *Arabidopsis*. Interestingly, ATX1 contains a PtdIns5*P* interaction domain (PHD finger) and thus, phosphoinositide signaling, may link environmental stress to changes in gene transcription.

**Principal Findings:**

Using the plant *Arabidopsis* as a model system, we demonstrate a link between PtdIns5*P* and the activity of the chromatin modifier ATX1 in response to dehydration stress. We show for the first time that dehydration leads to an increase in cellular PtdIns5*P* in *Arabidopsis*. The *Arabidopsis* homolog of myotubularin (AtMTM1) is capable of generating PtdIns5*P* and here, we show that AtMTM1 is essential for the induced increase in PtdIns5*P* upon dehydration. Furthermore, we demonstrate that the ATX1-dependent gene, *WRKY70*, is downregulated during dehydration and that lowered transcript levels are accompanied by a drastic reduction in H3K4me3 of its nucleosomes. We follow changes in *WRKY70* nucleosomal K4 methylation as a model to study ATX1 activity at chromatin during dehydration stress. We found that during dehydration stress, the physical presence of ATX1 at the *WRKY70* locus was diminished and that ATX1 depletion resulted from it being retained in the cytoplasm when PtdIns5*P* was elevated. The PHD of ATX1 and catalytically active AtMTM1 are required for the cytoplasmic localization of ATX1.

**Conclusions/Significance:**

The novelty of the manuscript is in the discovery of a mechanistic link between a chromatin modifying activity (ATX1) and a lipid (PtdIns5*P*) synthesis in a signaling pathway that ultimately results in altered expression of ATX1 dependent genes downregulated in response to dehydration stress.

## Introduction

Phosphoinositides can mediate differentiation, cellular growth, and responses to biotic and abiotic stress [1]. Differential phosphorylation of phosphatidylinositol results in phosphoinositides, which may function as precursors of second messengers or to modulate the localization, conformation, and activity of bound proteins. PtdIns5*P* is a minor component of the cellular lipid pool implicated in the cell osmoprotective response pathway [2], in the etiology of muscular and neuronal pathologies [3], and in the host-cell response to infection by *S. flexneri* [4]. In the nucleus, PtdIns5*P* can interact with ING2, an adaptor protein regulating p53 and histone acetylation in response to cellular stress [5], [6]. In plants, PtdInsPs function in responses to salinity, drought, and temperature stresses [7]–[9]. PtdIns5*P* accumulates in response to hyperosmotic stress in *Chlamydomonas*, in plant tissues from tomato, pea, alfalfa, and in cultured carrot cells [9]. PtdIns5*P* has not been formally identified in *Arabidopsis* [10] but *Arabidopsis* proteins Patellin1 and ATX1 can bind PtdIns5*P in vitro* [11], [12].

ATX1 is a chromatin modifier that tri-methylates lysine 4 of histone H3 of associated nucleosomes [13]. ATX1 binds specifically PtdIns5*P in vitro* and microarray analysis revealed a set of co-regulated genes [12], [14] suggesting the existence of a shared pathway in which PtdIns5*P* acts as a ligand modifying the activity of ATX1. However, evidence for the existence of PtdIns5P in *Arabidopsis*, for a change in the lipid level under stress, and for role of PtdIns5P in ATX1 activity is lacking. Here, we investigated whether PtdIns5*P* existed in *Arabidopsis*, whether the PtdIns5*P* level changed upon dehydration stress, whether endogenous PtdIns5*P* could affect the activity of ATX1, and how this might occur. We tested the hypothesis that dehydration stress increases cellular PtdIns5*P*, which acting as a ligand modulates ATX1 activity *in vivo*. Without excluding a role for PtdIns5*P* in chromatin [15], we elucidate a PtdIns5*P*-driven mechanism that sequesters ATX1 in the cytoplasm suppressing its function in the nucleus. The cytoplasmic sequestration is dependent on the ePHD finger, which interacted with PtdIns5*P in vitro*, and on factors that affect the cellular level of PtdIns5*P*. The model suggests a pathway mediated by PtdIns5*P* that translates environmental stress into altered activity of a chromatin modifier.

## Results

### PtdIns5P in Arabidopsis thaliana

**Figure 1:**
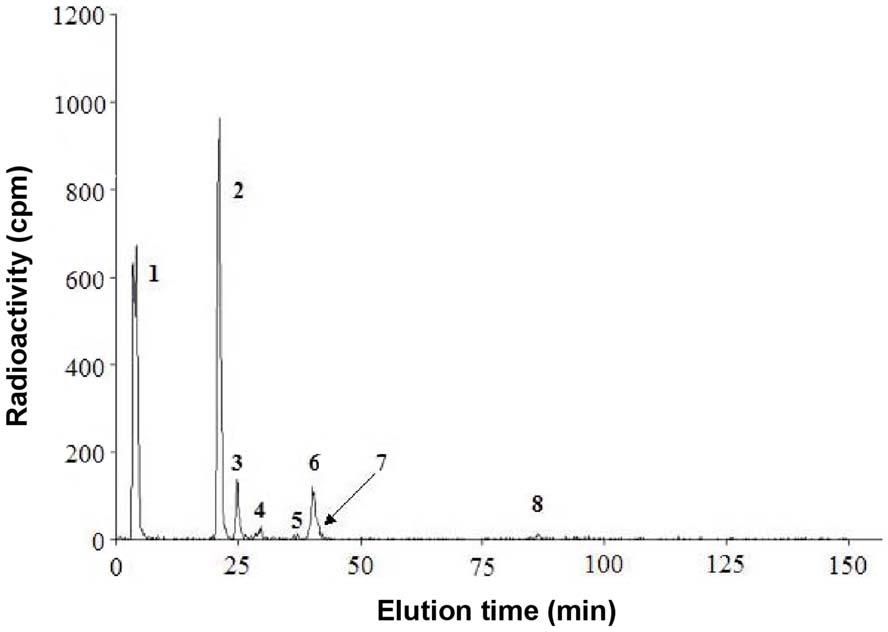
Failure to detect PtdIns5P by HPLC. HPLC analysis of deacylated lipid products isolated from rosette leaves of *in vivo* radiolabeled whole plants with orthophosphate (300 µCi/ml) for 16 hours at room temperature. Peak identification: 1, phospholipids with positive charge; 2, phosphatidylinositol; 3, phosphatidic acid; 4, free inorganic phosphate; 5, PtdIns3*P*; 6, PtdIns4*P*; 7, PtdIns5*P*; 8, PtdIns(4,5)*P*_2_.

A high cellular level of PtdIns4*P*, a predicted low level of PtdIns5*P*, and the overlap of PtdIns4*P* and PtdIns5*P* peaks in high performance liquid chromatography (HPLC) impede identification of PtdIns5*P* in *Arabidopsis* [10] (Figure 1).

To determine the presence of endogenous PtdIns5*P* in *Arabidopsis*, we used a mass assay based on the specificity of PIP4Kα to phosphorylate PtdIns5*P* at the 4′-position of the inositol head group [16] allowing a quantitative determination of intracellular PtdIns5*P* [17].

**Figure 2:**
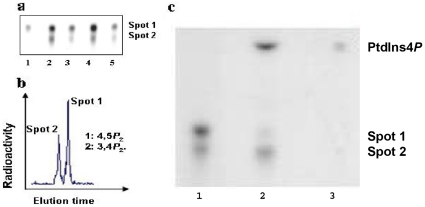
PtdIns5*P* in *Arabidopsis* tissues determined by the PIP4Kα assay. Thin layer chromatographic separation of the products of the *in vitro* phosphorylation reaction between PIP4Kάandendogenous phosphoinositides isolated from leaves (lane 2), stems (lane 3), flowers (lane 4) and siliques (lane 5). A PtdIns(4,5)*P*_2_ standard is shown (lane 1) (panel a). HPLC analysis of deacylated products of the *in vitro* phosphorylation reaction between endogenous phosphoinositides (from leaves) and PIP4Kα. Deacylated PtdIns(3,4)*P*_2_ and PtdIns(4,5)*P*_2_ used as standards (panel b). The products of the *in vitro* phosphorylation reaction between endogenous phosphoinositides isolated from rosette leaves and PIP4Kα were incubated in the absence (lane 1) or presence (lane 2) of yeast YNK-5 phosphatase. The reaction products were analyzed by thin layer chromatography. The position of a PtdIns4*P* standard is shown (lane 3) (panel c).

Total phospholipids extracted from four different tissues were used as starting material for the PtdIns5*P* mass assay. The two labeled PtdIns*P*_2_ isomers formed in all the reactions were putatively identified as PtdIns(4,5)*P*_2_ (spot 1) and PtdIns(3,4)*P*_2_ (spot 2) by thin layer chromatography (TLC) (Figure 2a). According to the known substrate specificity of PIP4Kα [16], these reaction products reflected the endogenous PtdIns5*P* and PtdIns3*P*, respectively. To further identify the products they were deacylated, subjected to HPLC analysis and identified as PtdIns(4,5)*P*_2_ and PtdIns(3,4)*P*_2_ (Figure 2b). In all subsequent analyses we focused exclusively on determining the mass of endogenous PtdIns5*P*.

Both TLC and HPLC analyses indicated presence of PtdIns5*P* in the four tested plant tissues: rosette leaves, stems, inflorescences, and siliques. However, a potential caveat was that although PtdIns4*P* is not a substrate for the PIP4Kα, it was still possible that the enzyme had different substrate specificity for PtdIns*P*s of plant origin, particularly in cases when PtdIns4*P* is more abundant than PtdIns5*P*. To confirm that PIP4Kα phosphorylated PtdIns5*P* on the 4′ position of the inositol ring, the two radiolabeled products (spots 1 and 2) were together incubated in the absence or presence of yeast 5′-phosphatase (Figure 2c).

A decrease in the radioactivity in the PtdIns(4,5)*P*_2_ spot coinciding with the appearance of radioactivity in PtdIns4*P* (containing an equal amount of radiolabel lost from PtdIns(4,5)*P*_2_ (spot 1) indicated that the radiolabel in the PtdIns(4,5)*P*_2_ was exclusively on the 4′-position. The 5′-phosphatase did not affect the intensity of the PtdIns(3,4)*P*_2_ (spot 2). We conclude that the PtdIns(4,5)*P*_2_ produced by PIP4Kα originates from endogenous PtdIns5*P* present in *Arabidopsis*.

### Cellular PtdIns5*P* levels upon osmotic and hypotonic stresses

Osmotic stress increases PtdIns5*P* in yeast, animal, and plant cells [2], [9], [10], [18]. Dry air, drought and floods are environmental factors that stimulated our interest to examine changes in cellular PtdIns5*P* upon exposure of *Arabidopsis* to these stresses. In concurrent unrelated studies we have established that 9–12 days exposure to water withdrawal conditions (in-soil dehydration stress) followed by 3 day-watering resulted in cellular water loss (∼60% residual water levels) and cellular water level recovery (as measured by the restoration of cell turgidity) that were similar to the water-loss and recovery levels achieved in detached leaves when exposed for 2 hours at room temperature conditions (20°C) followed by an overnight submergence in water [19; a submitted manuscript]. In addition, air-dried detached leaves showed little variation in residual cellular water levels (between 58–62%) making it the preferred choice for inducing dehydration stress in experiments exploring cell responses in four different backgrounds and under different conditions as described here.

**Figure 3:**
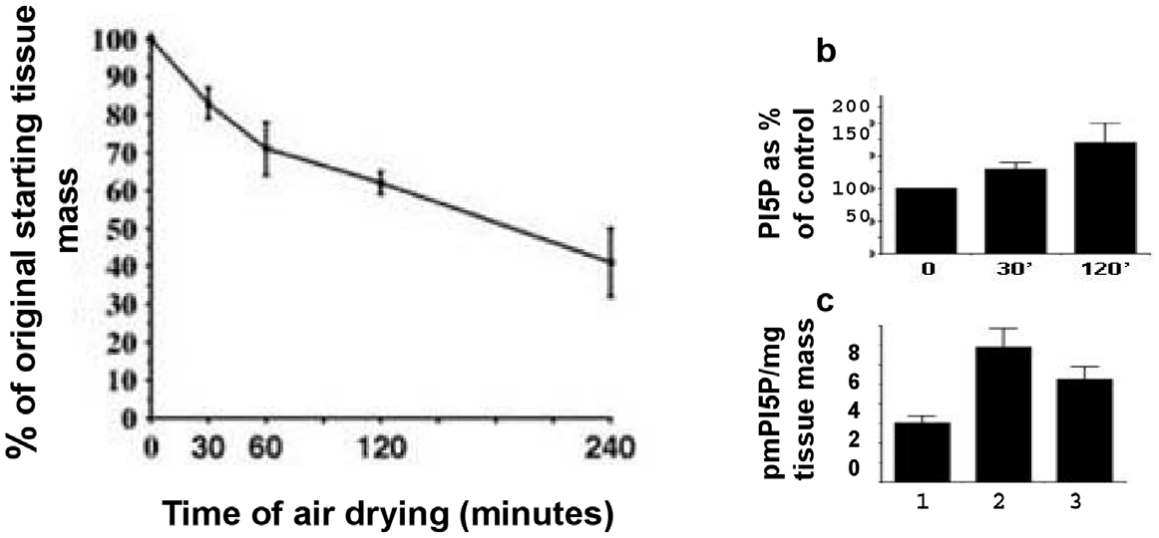
Time-course of water loss from detached leaves. Water-loss in detached rosette leaves after exposure to ambient air and a temperature of 20°C determined as percentage of residual tissue mass taken as 100%. Data are from four independent experiments; bars are s.d. (panel a). Lipids extracted from leaves after 30 minutes or 120 minutes of air-exposure were processed for PtdIns5*P* content and expressed as the percentage of the fresh sample (indicated as 100%). Data are from six independent experiments; bars are s.d. (panel b). Detached (column 1), air-exposed (column 2) and water-submerged leaves (column 3) were treated in parallel for 120 minutes. Cellular PtdIns5*P* is indicated as pmoles PtdIns5*P*/mg initial fresh tissue mass. Bars are s.d. (panel c).

**Figure 4:**
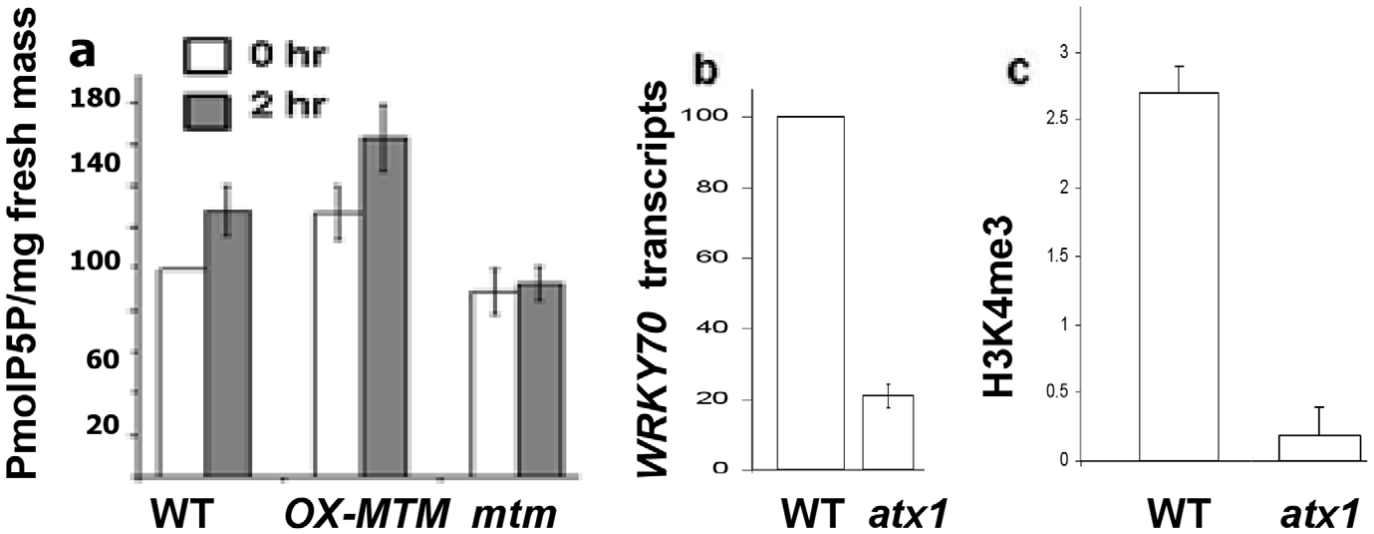
PtdIns5*P* levels and ATX1 activity in *AtMTM1* mutant cells. PtdIns5*P* levels in fresh and dehydrated wild type leaves, in OX-AtMTM1 leaves, and in leaves from homozygous *mtm1* plants (panel a). Relative *WRKY70* transcripts in non-stressed and in stressed wild-type, OX-AtMTM1, and *mtm1* mutant cells, quantified by real-time PCR (panel b). Quantitative PCR of ChIP assays of H3K4me3 methylation in wild-type, *OX-ATMTM1* and *mtm1* mutant chromatins in fresh leaves and in leaves after dehydration-stress (panel c). Relative enrichment in *WRKY70*-specific fragments *vs* input DNA.

Optimal conditions for our analyses were established by harvesting rosette leaves and exposing them to ambient air (20°C). Water-loss was determined as the residual tissue mass measured up to four hours of air-exposure (Figure 3a). PtdIns5*P* was measured in total lipids extracted after 30 minutes and after 2 hours exposure to air. Leaves collected from the same plants subjected immediately to lipid extraction were used as controls (0 min). In fresh (non-treated) *Arabidopsis* leaves, the amount of total phospholipid and of PtdIns5*P* measured in six independent experiments were: 2.08 nmoles/mg fresh tissue (s.d. 0.22) and 3.6 pmoles/mg fresh tissue (s.d. 0.096), respectively (Figure 3b, see also Figure 4a). To examine the effect of water stress, detached leaves were submerged in water for 2 hours before measuring lipid content. Leaves collected from the same plants and immediately subjected to lipid extraction were used as controls (Figure 3c). The results suggested that leaf submergence in water also increased the cellular PtdIns5*P*, although to a lesser degree than dehydration for the same length of time. Because a 2 hour exposure to air showed the highest level of PtdIns5*P* accumulation, we used this condition (from here on referred to as dehydration-stress) to experimentally trigger elevated cellular PtdIns5*P*.

### PtdIns5*P* levels in AtMTM1 over-expressing and knockout cells

A family of phosphoinositide 3′-phosphatases (myotubularins, MTM1s) generates cellular PtdIns5*P* through their activity towards PtdIns3,5*P*_2_ [20]–[22]. Inactivation or constitutive expression of MTM1 is a strategy used to study the roles of its substrates (PtdIns3*P* and PtdIns3,5*P*_2_) and respective products (PI and PtdIns5*P*) in cellular homeostasis.

Recently, we found that the *Arabidopsis* gene *At3g10550* encodes a protein 59% similar to the human MTMR2 (called *AtMTM11*) and, like mammalian myotubularins, has a phosphoinositide 3′-phosphatase activity against both PtdIns3*P* and PtdIns3,5*P*_2_ [19]. Whether constitutive expression of AtMTM1 could increase the level of cellular PtdIns5*P* levels in plant cells is a key question, which was addressed by over-expressing AtMTM1 (*OX-AtMTM1*) in transgenic plants. The cellular PtdIns5*P* level was determined in transgenic and wild type plants before and after stress. Differences in PtdIns5*P* levels were evaluated by the Mann-Whitney (U)-test [23].

Upon dehydration, PtdIns5*P* increased by ∼30% in wild type leaves (U-test p<0.006, Figure 4a). A further increase was seen upon dehydration of OX-AtMTM1 cells producing ∼70% more PtdIns5*P* than non-stressed wild type cells (U-test p<0.002) implicating AtMTM1 in elevating cellular PtdIns5*P* upon stress. Importantly, solely the over-expression of AtMTM1 led to an increase in PtdIns5*P* that was similar in magnitude to dehydration stress stimulated wild type plants. To assess if AtMTM1 links dehydration stress with increased PtdIns5*P*, we measured the endogenous level of PtdIns5*P* in homozygous knockout *mtm1* mutant plants. In non-stressed *mtm1* leaves, PtdIns5*P* was not significantly different from that in non-stressed wild type leaves (Figure 4a, U-test p>0.2). However, the increased PtdIns5*P* seen in wild type plants in response to stress was completely blunted in *mtm1* mutant leaves producing significantly less PtdIns5*P* (∼35% less, U-test p<0.01) than wild type leaves under stress. The difference was even more pronounced when dehydration-stressed *mtm1* mutant leaves were compared with dehydration stressed OX-AtMTM1 leaves (∼75% less PtdIns5*P*, U-test p<0.001) (Figure 4a). These data underscore the role of MTM1 activity in increasing PtdIns5*P* upon stress but not of the basal PtdIns5*P* level. As both PtdIns5*P* and ATX1 regulate *WRKY70* and ATX1 interacts with PtdIns5*P*, we sought to determine a mechanistic link between these observations in response to dehydration stress.

### ATX1 activity in stressed plants

**Figure 5:**
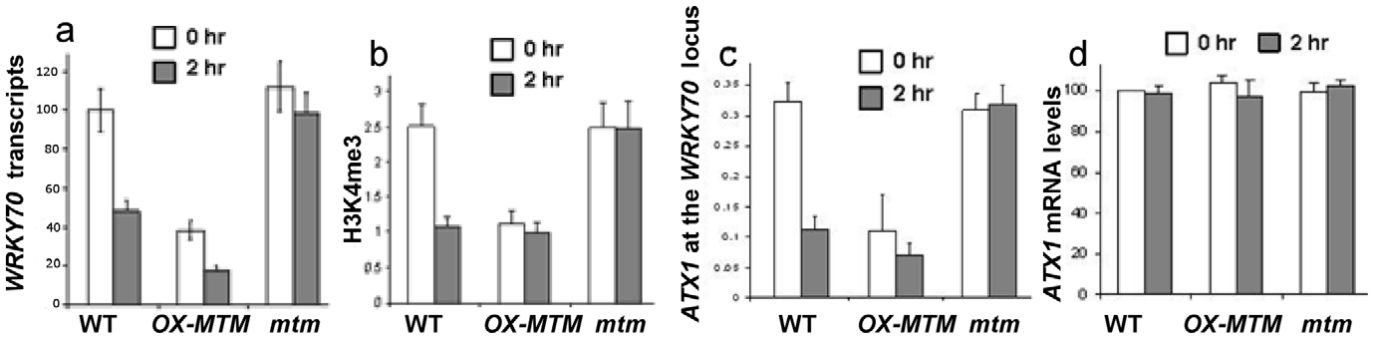
ATX1 activity under drought conditions and different backgrounds. Relative *WRKY70* transcripts in freshly harvested leaves and in leaves after 2 h air-exposure in wild type, *OX-AtMTM1*, and *mtm1* backgrounds quantified by real-time PCR normalised *versus* actin (panel a). Quantitative PCR of ChIP assays of *WRKY70* H3K4me3 levels in wild type control and dehydration-stressed tissue and in respective samples in the *OX-AtMTM1* and *mtm1* backgrounds. The y-axis represents the relative enrichment of recovered DNA *versus* the input (panel b). Quantitative PCR ChIP assay with anti-ATX1 antibodies at the *WRKY70* locus before and after dehydration-stress in wild type, *OX-AtMTM1*, and *mtm1* mutant chromatins; relative enrichment in *WRKY70*-specific fragments *vs* input DNA (panel c). Relative *ATX1* transcript levels in freshly harvested leaves and in leaves after 2 h air-exposure in wild type, *OX-AtMTM1*, and *mtm1* backgrounds quantified by real-time PCR normalised *versus* actin (panel a). Bars are s.d.

As previously shown [24] the transcript level of *WRKY70* is regulated by ATX1 as it decreased in homozygous *ATX1* deleted (*atx1*) plants (Figure 4b). Chip analysis with antibodies specific to tri-methylated histone 3 lysine4 showed a dramatic decrease in H3K4me3 signal from the 5′ end of the *WRKY70* gene in *atx1* plants (Figure 4c), showing that the activity of ATX1 is largely responsible for controlling the levels of H3K4me3 at the *WRKY70* promoter. Furthermore, and important to this study, the level of *WRKY70* transcript was decreased in response to dehydration stress (Figure 5a). Previous microarray analyses have suggested that PtdIns5*P* negatively influences ATX1 activity [12]. Whole-genome analyses, however, could not provide answers to two critical questions: whether increased PtdIns5*P* affected the H3K4-methylation profiles of ATX1 targets and whether association of ATX1 with target nucleosomes changed under elevated PtdIns5*P*. Here, we use the *WRKY70* gene as a model to investigate ATX1 activity *in vivo* under dehydration-stress or under experimental conditions where we manipulated the levels of endogenous PtdIns5*P*. Selection of the *WRKY70* gene was determined by three important factors: the *WRKY70* nucleosomes are directly modified by ATX1 activity [24], its transcript levels are subject to regulation by dehydration stress (Figure 5a), and *WRKY70* transcript levels were diminished upon addition of exogenous PtdIns5*P* [12].

### *WRKY70* expression and H3K4me3 profiles in *OX-AtMTM1* and in *mtm1* mutant cells

Similar to the results with dehydration-stressed wild type leaves, lower *WRKY70* transcripts were found in stressed OX-AtMTM1 leaves (Figure 5a). However, the levels of *WRKY70* transcripts in basal conditions were already significantly decreased and similar to the levels measured in wild type plants after dehydration stress, correlating with the increased levels of PtdIns5*P* (Figure 5a). By contrast, in non-stressed *mtm1* cells, the *WRKY70* transcripts were not significantly different than in the wild type (p<0.12) but, importantly, the decrease in *WRKY70* transcripts in response to the stress was suppressed (Figure 5a). Consistent with the level of the *WRKY70* transcripts there was less H3K4me3 at the *WRKY70* nucleosomes in basal *AtMTM1*-overexpressing plants, similar to the levels seen in dehydration stressed wild type plants (Figure 5b). In *mtm1* plants where the increased PtdIns5*P* is abrogated in response to dehydration stress, the H3K4me3 levels were not significantly changed after stress (Figure 5b). These data show that the transcript levels of *WRKY70* and the H3K4me3 patterns of its nucleosomes correlate with manipulated cellular PtdIns5*P* levels.

**Figure 6:**
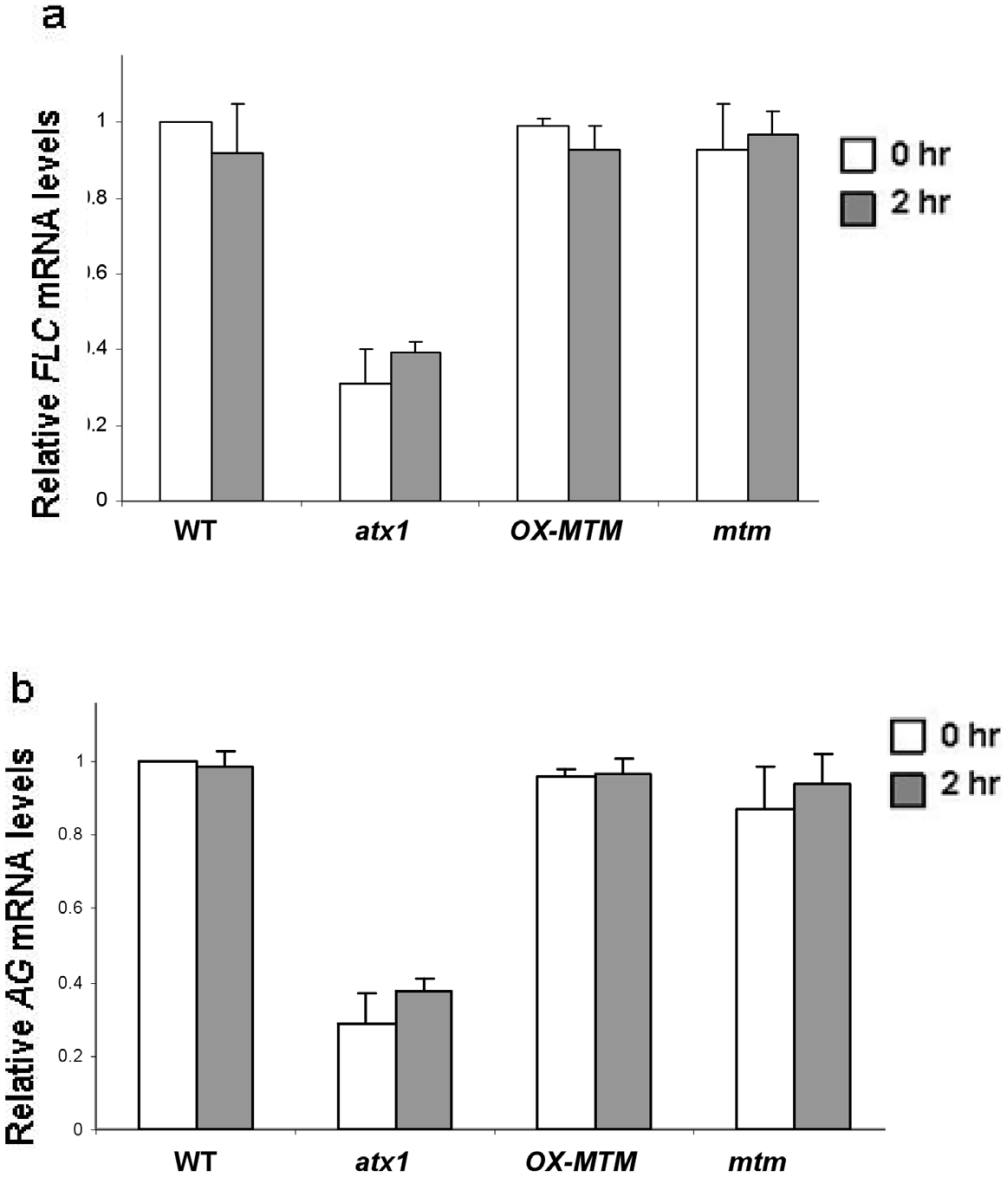
*FLC* and *AG* transcripts are not regulated by AtMTM activity. Relative *FLC* transcripts determined in leaves of 12 day old seedlings in non-stressed and in stressed wild-type, *atx1*, *OX-AtMTM*, and *mtm* mutants, quantitated by real-time PCR and normalized against actin (panel a). Relative *AG* transcripts in inflorescences of wild-type, *atx1*, *OX-AtMTM*, and *mtm* mutants, under non-stressed and stressed conditions (panel b). Bars are s.d.

To mechanistically link changes in the H3K4me3 patterns, we assessed the localization of ATX1 at the *WRKY70* promoter using the ChIP assay with anti-ATX1 antibodies. ATX1 was localized at the *WRKY70* 5′-end but its presence decreased in response to dehydration stress of wild type plants. In non-stressed plants that over-express *AtMTM1* the levels of ATX1 were already significantly decreased and reflected levels seen in stressed wild type plants. In contrast, in *mtm1* knock out plants, ATX1 localized at the *WRKY70* but was unaltered in response to dehydration stress (Figure 5c). These data suggest that transcription of *WRKY70* is regulated in response to dehydration stress in a PtdIns5*P* dependent manner. Changes in PtdIns5*P* appear to control the localization and the activity (H3K4me3) of ATX1 at the promoter. These changes are not a reflection of changes in ATX1 expression as it was not altered by stress nor was it altered in the various genotypes (Figure 5d). These results illustrated that the ATX1 level at the *WRKY70* gene locus changed in response to dehydration and depended on the presence of a functional AtMTM1. Decreased presence of ATX1 at the *WRKY70* gene correlated with decreased transcript and H3K4me3 levels (Figure 5a–c). By contrast, transcript levels from two other ATX1 direct targets (*AG* and *FLC*) not involved in the dehydration-stress response and not found in the PtdIns5*P*-ATX1 co-regulated set [12] did not change upon stress in the *OX-AtMTM1* or *mtm1* backgrounds (Figure 6).

### Cellular distribution of ATX1

**Figure 7:**
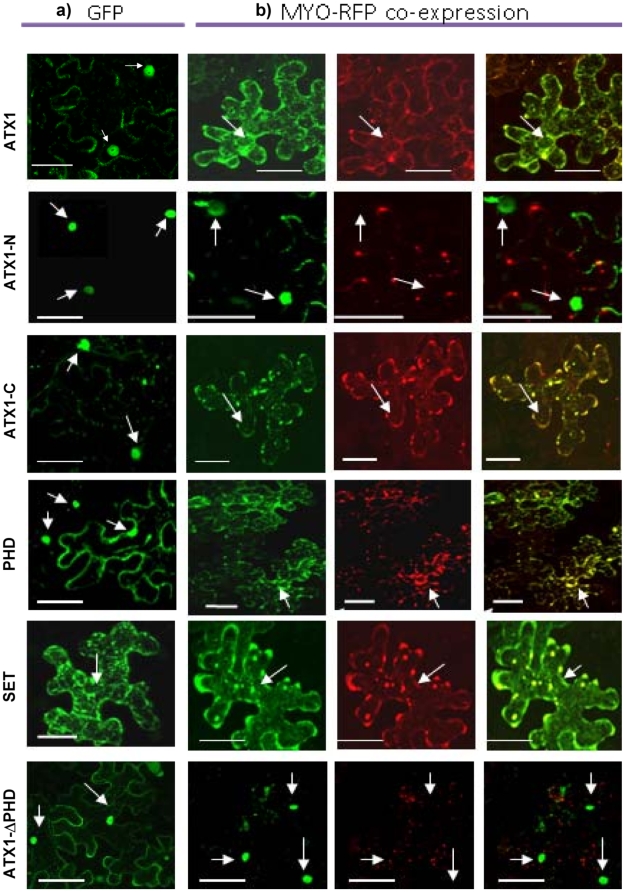
Sub-cellular distribution of ATX1 co-expressed with AtMTM1. ATX1 and its derivatives were expressed as ATX1-GFP fusion proteins (green signal) while AtMTM1 was expressed as an RFP fusion protein (red signal). **a)** all images in this column illustrate cells expressing ATX1 or derivatives alone; **b)** all images in this column illustrate cells co-expressing ATX1 (or ATX1 derivatives) with AtMTM1. Arrows point to nuclei in cells expressing a GFP-fusion protein. ATX-N is the N-terminal ATX1 portion, ATX1-C is the C-terminal ATX1 portion containing the ePHD and the SET domains (SF7). Arrows point to nuclei; Bars are 50 µm.

**Figure 8:**
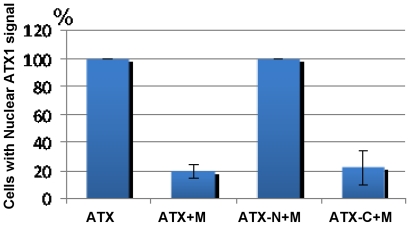
Cells showing nuclear GFP-signal associated with ATX1 protein or its derivatives. The number of cells showing nuclear localization of transiently expressed ATX1 alone is set at 100%. ATX+M represents the population of cells displaying nuclear signal when co-expressing the entire ATX1 and AtMTM; ATX-N+M represents cells co-expressing the entire N-terminal portion of ATX1 and AtMTM; ATX-C+M are cells co-expressing the ATX1-C-terminal portion and AtMTM, respectively; bars are s.d. from four independent experiments.

ATX1 can bind PtdIns5*P* [12] and here we analyze the cellular localization of ATX1 under conditions that elevated cellular PtdIns5*P*. Dehydration raises PtdIns5*P* but causes partial collapse of internal structures (not shown) complicating cytological analysis. As an alternative, distribution of GFP-tagged ATX1 (wild-type or various deletion forms) was followed in the presence of AtMTM1-RFP-fusion protein co-expressed in tobacco leaf-epithelial cells. ATX1-GFP is seen in the nuclei, in the cytoplasm, and at the plasma membrane (Figure 7a). However, in cells co-expressing ATX1 and AtMTM1 most of the ATX1 was absent from the nuclei (Figure 7b). Quantitation showed that ATX was present in the nuclei when expressed alone but when co-expressed with AtMTM1, its presence in nuclei decreased to about 20% (Figure 8).

**Figure 9:**
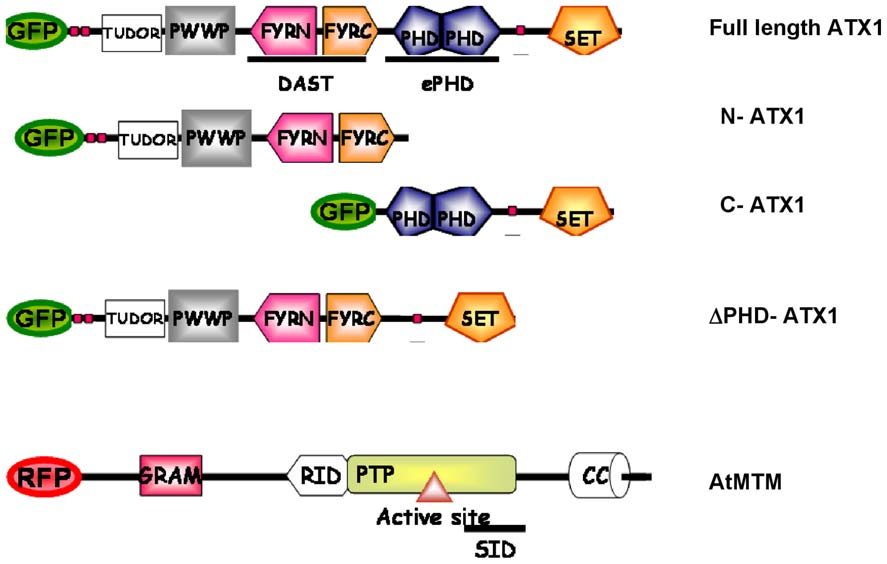
Structure of representative constructs used in the study. The FYRN and FYRC domains are called DAST, for Domain Associated with SET in Trithorax; the ePHD belongs to a distinct, extended PHD, family of proteins. The putative AtMTM domains are labeled according to their homology to the human MTMR2.

Next, we determined which of the ATX1 structural domains was involved in the re-localization. The N-terminal portion of ATX1 (Figure 9) localizes in the nucleus (Figure 7a). Over-expression of AtMTM1 did not relocate the nuclear ATX1 (Figure 7b) suggesting that the N-terminal protein half was not involved. When the C-terminal portion of ATX1 was expressed alone, it localized in both the nuclei and in the cytoplasm; however, the nuclear signal decreased in cells co-expressing AtMTM1 (Figures 7a, b and Figure 9) implicating it in the re-localization. The ATX1-C terminal portion contains the ePHD and the SET structural domains (Figure 9), which were expressed separately to determine their individual roles. The SET domain expressed alone, or co-expressed with AtMTM1, remained in the cytoplasm (Figure 7a, b) implying that the SET domain was not involved in the ATX1 nuclear localization. However, the ePHD-GFP, which was present in the nucleus when expressed alone, became cytoplasmic when co-expressed with AtMTM1-RFP (Figure 7a, b) suggesting that the ePHD is involved in the nucleus-to-cytoplasm re-localization of ATX1 in cells over-expressing AtMTM1.

The role of the ePHD in the cytoplasmic retention of ATX1 was proven further by generating an ATX1 protein with deleted ePHD. When expressed alone, ATX1- ΔPHD-GFP localized in the nucleus; however, in cells co-expressing MYO-RFP, the GFP signal remained nuclear contrasting the redistribution seen with wild-type ATX1-GFP (Figure 7a, b). We conclude that the ATX1-ePHD is involved in the subcellular localization of ATX1 likely through binding the ligand PtdIns5*P*.

### PtdIns5*P* and the role of ePHD

**Figure 10:**
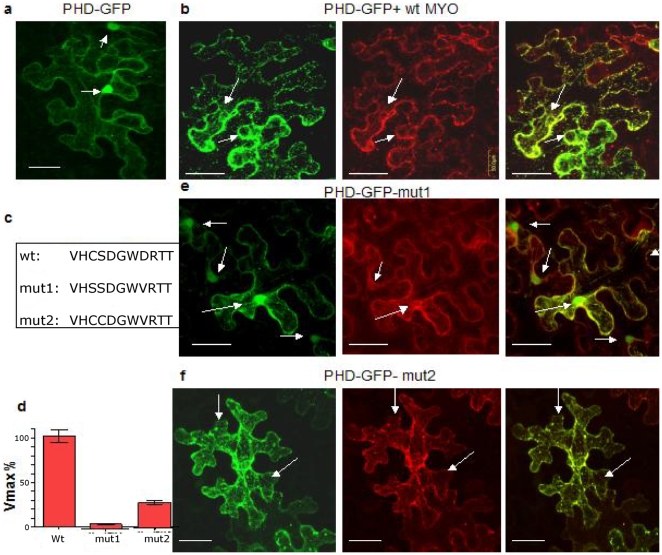
PtdIns5*P* in the cytoplasmic retention of ePHD is dependent on AtMTM1 catalytic efficiency. Nuclear localisation of ePHD-GFP expressed alone. Arrows in all panels point to nuclei (panel a). ePHD-GFP co-expressed with wild-type AtMTM1-RFP causes depletion of the green nuclear signal (panel b). The consensus amino acids in the active AtMTM1 phosphatase site and the amino acid substitutions in *mut1* and *mut2* (panel c). Phosphoinositide 3′-phosphatase activity of recombinant wild type, *mut1* and *mut2* phosphatases. The Vmax values towards PtdIns(3,5)*P*_2_ as a substrate are shown as the percentage of the wild-type (WT) ATMTM1 activity. Bars are s.d. from three measurements (panel d). Distribution of ePHD-GFP co-expressed with mut1AtMTM1-RFP show nuclear localizations of the green signal (panel e). Co-expression of ePHD-GFP with mut2AtMTM1-RFP. The green signal is largely distributed in the cytoplasm (panel f). Bars are 50 Pm.

Two mutually non-exclusive mechanisms may account for the cytoplasmic retention of ePHD in OX-AtMTM1 cells: elevated PtdIns5*P* binds and keeps the protein in the cytoplasm and/or the AtMTM1 protein binds the ePHD. The ability of ePHD_ATX1_ to specifically bind PtdIns5*P in vitro* supports the first model [12]. To provide evidence *in vivo*, we generated AtMTM1 isoforms with a mutant active site to test whether production of PtdIns5*P* by AtMTM1 is required for the cellular localization of the ePHD. A protein with a mutation in the critically important Cys in the active site (*mut1*) displayed low (∼5%) phosphatase activity against its substrate PtdIns3,5*P*_2_ compared to the wild type. A second protein with a mutation in the adjacent Ser (*mut2*) retained ∼30% of wild type activity (Figure 10c, d). Co-expression of ePHD with mut1-RFP failed to shift ePHD from the nucleus to the cytoplasm (Figure 10a, e) in contrast to experiments with the wild-type AtMTM1-RFP (Figure 10a, b). Importantly, co-expression of ePHD with mut2-RFP resulted in a distribution similar to the one observed with wild-type AtMTM1-RFP (Figure 10b, f). Because the effects of the AtMTM1 mutants on the cellular distribution of ePHD correlate with their lipid 3′-phosphatase activity we conclude that production of PtdIns5*P* is required for retaining ePHD in the cytoplasm. These results do not preclude binding of ePHD and AtMTM1, a possibility that remains to be explored.

## Discussion

By analyzing the H3K4me3 pattern of the *WRKY70* nucleosomes, we followed changes in ATX1 activity *in vivo* under stress or in the presence of overexpressed AtMTM1. The statistically significant decrease in *WRKY70* nucleosomal H3K4me3 in dehydrated wild type cells, in parallel with an increase in cellular PtdIns5*P*, reflected diminished ATX1 activity. Increasing cellular PtdIns5*P* by overexpressing AtMTM1 also led to a diminution of ATX1 activity and decreased H3K4me3 of *WRKY70*. Attenuation of dehydration stress-induced PtdIns5*P* generation in *mtm1* mutant cells attenuated also the dehydration stress induced changes in H3K4me3 and transcription of *WRKY70*. Under all of these conditions, changes in H3K4me3 and *WRKY70* transcripts correlated with the presence of ATX1 at the promoter nucleosomes, as assessed by ChIP (Figure 5c), suggesting that PtdIns5*P* may regulate ATX1 localization. The cytoplasmic localization of ATX1 observed under increased cellular PtdIns5*P* was in accordance. The endogenous PtdIns5*P* level was not significantly lower than in the wild type suggesting that PtdIns5*P*/PtdIns3,5*P_2_* homeostasis was not severely disrupted in AtMTM1-depleted cells. Under stress, however, the *mtm1* mutant cells failed to elevate PtdIns5*P* underscoring the role of AtMTM1 in producing PtdIns5*P*. These data are consistent with a causal role for a dehydration-induced PtdIns5*P* regulating the localization of ATX1. Furthermore, H3K4me3 and transcripts from *WRKY70* did not change significantly in *mtm1* cells indicating a requirement for PtdIns5*P*. These results, however, do not preclude an interaction of AtMTM1 with ATX1-SET. An intriguing feature of all known active and inactive myotubularins, including the two *Arabidopsis* proteins, is the presence of a conserved domain, SID (Set interacting domain) that specifically binds the SET [25] domain. The SID domain is conserved in all known active and inactive members of the myotubularin family [25], [26]. The biological significance of this interaction, however, is under debate, as crystallographic studies have shown that the SID peptide is buried inside the tertiary structure of the protein arguing against its binding with other proteins [27]. However, mutations in SID impeding its binding to SET result in abnormal growth and leukemia [25], [28] and the inactive myotubularin Sbf1 has been isolated as a component of the Trx complex, physically and functionally linked with the trithorax protein in *Drosophila* [29]. An important implication of these results is that myotubularins, active or inactive, may have nuclear localization and could participate in chromatin remodeling [30].

Lack of a visible phenotype in *mtm* mutants and the level of endogenous PtdIns5*P* not significantly lower than in fresh wild type cells suggest that the AtMTM1 function is not essential for the cellular homeostasis in non-stressed cells, in stark contrast with humans where myotubularin deficiency is implicated in severe muscle dystrophy, neuronal diseases, and leukemia [1], [3], [31]. Despite highly conserved in evolution, molecular tools are adapted and used in kingdom and species-specific manners [32]. Two phosphatases that can convert PtdIns(4,5)*P*_2_ to PtdIns5*P* were discovered in human cells [33] but whether a similar route for generating PtdIns5*P* exists in plants is unknown.

As a substrate of AtMTM1, it is possible that PtdIns3*P* and PtdIns(3,5)*P*_2_ cellular levels were also affected. PtdIns3*P* is associated with late endosomes, multivesicular bodies and pre-vacuolar membranes [8] suggesting a possible indirect involvement of PtdIns3*P* in the reported events. However, the ability of the ePHD finger to bind PtdIns5*P* suggests that PtdIns5*P* is likely to be the endogenous regulator of ATX1 localization.

Most likely, AtMTM1 has a broader role than just regulating ATX1 consistent with our results from whole-genome transcription profiling of PtdIns5*P*-treated [12] and of *OX-MTM1* genomes [19]. Our data suggest that ATX1 and AtMTM1 (PtdIns5*P*, respectively) function in distinct pathways, which partially overlap only along branches that target a shared set of genes. In agreement, ATX1 regulated stress-response genes that are not involved in the PtdIns5*P/*MTM1-pathway (i.e. the *NCED3* gene, not shown); the developmentally regulated *AG* and *FLC* genes (Figure 6a, b) are efficiently transcribed during dehydration stress, illustrating different patterns of ATX1 activity, and perhaps, regulation. The dynamic subcellular localization of ATX1 in non-stressed root cells [12] suggested that, most likely, ATX1 was responding to a variety of cellular signals. Furthermore, the *FLC* gene, which is under the control of complex genetic, epigenetic, and growth-signaling mechanisms, re-establishes anew the H3K4me3 modification pattern at the transcription start site in each developmental cycle. H3K4me3 levels correlate with reactivated *FLC* transcription, which persists until transition to flowering [34]. It is plausible, then, that the *FLC* H3K4me3 and transcript levels established early in development might not be affected by ATX1 presence at the *FLC* locus later in life. On the other hand, the *WRKY70* gene displaying dynamic H3K4me3 changes in responses to biotic [24] and dehydration stresses (this study) showed a clear dependence on ATX1 presence.

Production of PtdIns5*P* is required for retention of ePHD in the cytoplasm (Figure 10). The ePHD found in all animal and plant members of the Trithorax family belongs to a family distinct from the families of the PHD domains of the chromatin proteins ING, NURF, CHD, ORC1, or ACF. Some PHDs bind PtdIns5*P* [5], [6] as well as H3K4me3 [35], [36]. Whether ePHD binds histones is unknown and a role for ePHD at chromatin is not excluded. Here, we report a novel function for the ePHD domain of a chromatin-associated protein as a mechanism regulating ATX1 activity. The shared ATX1-AtMTM1 dehydration-responding fraction identified by microarray analysis provided further evidence for a pathway involving an epigenetic regulator and a lipid phosphatase producing PtdIns5*P* (microarray expression data are deposited in the NCBI series GSE15577). The genes co-regulated by ATX1 and by exogenously supplied PtdIns5*P* [12] further support the biological relevance of the ATX1 binding to this lipid ligand. A bound lipid may activate or deactivate the protein by sequestering it in a cellular sub-compartment, by inducing a conformational change, or by triggering phosphorylation of another downstream target [37]. It is possible that PtdIns5*P* can regulate ATX1 activity in both nuclear and cytoplasmic contexts and that different mechanisms may operate in the two cellular compartments.

## Materials and Methods

### Plant material

Wild type and mutant plants were grown in soil under the same controlled daylight environmental conditions. Leaves were detached from plants and immediately weighed to determine their fresh weight. Recovery upon dehydration was established by measuring turgidity as described in [38]. Briefly, dehydration stressed leaves were submerged in deionized water for 24 h, blotted dry and weighed to determine their TW (turgid weight).

Transgenic plants were generated by transformation with binary vectors. Binary plasmids were transformed into chemically competent *Agrobacterium tumefaciens* strain C58C1 by incubating DNA with *Agrobacteria* on ice for 5 minutes, freezing in liquid nitrogen for 5 minutes and heat shock at 37°C for 5 minutes. The cells were allowed to recover in growth medium with shaking for 2 hours at room temperature and plated on selection medium containing rifampicin, gentamycin and a third antibiotic for plasmid selection. *Agrobacteria* selected for transformation were used to transform Col-O plants using a floral dipping method as described [39].

To generate stably *AtMTM*-over-expressing lines the full-length sequence of *At3g10550* was amplified using PCR-specific primers containing the *attB1* and *attB2* sequences and recombined by the attB x attP (BP) reaction into the pAlligator vector [40] to generate an N-terminal HA-tagged expression clone for stable expression in plants. The entry clone was also recombined into the pB7WGR2.0 vector [41] to generate an N-terminal RFP fusion expression clone for expression in plants. The binary vector pAlligator2-AtMTM construct containing the coding sequence of the full length *AtMTM* gene cloned in-frame with an N-terminal HA tag and driven by the 35S promoter was used for transformation in *Arabidopsis*. Transgenic plants were selected by GFP-fluorescent seeds where expression of the GFP marker was driven by a seed-specific promoter. The T-DNA insertion line SALK_029185 was obtained from the SALK institute. Homozygous (*mtm*) lines were screened by segregation, PCR, and RT-PCR analyses; *atx1* mutant lines were as described [13].

### Constructs

The following gateway specific PCR products were obtained as follows:

*ATX1-Full Length cDNA* was PCR amplified using forward primer *atx1attB1*:

5′-GGGGACAAGTTTGTACAAAAAAGCAGGCTTAATGGCGTGTTTTTCTAACGAAAC-3′ and the reverse primer *atx1-attB2*: 5′-GGGGACCACTTTGTACAAGAAAGCTGGGTATTCTGCGGTCCAGTCTATTAGAT-3′.

*The N-terminal domain* of ATX1 was PCR amplified using the forward primer *atx1attB1* and the reverse primer *dast-attB2*:

5′-GGGGACCACTTTGTACAAGAAAGCTGGGTAATCGAGATCTTTCCAGTCAACAC-3′.

*The C-terminal domain* of ATX1 was PCR amplified using the forward primer *phd-attB1*:

5′-GGGGACAAGTTTGTACAAAAAAGCAGGCTTAAAGTGCAATGTCTGCCAC-3′ and the reverse primer *atx1-attB2*.

*The ePHD domain* of ATX1 was PCR amplified using the forward primer *phd-attB1* and the reverse primer *atx1-attB2*.

*The SET* domain of ATX1 was PCR amplified using the forward primer set-attB1

5′-GGGGACAAGTTTGTACAAAAAAGCAGGCTTAATGAATACTCCAAGCAAC-3′ and the reverse primer *atx1-attB2*.

The gateway PCR products were cloned into the entry vector pDONR221 and the entry clones recombined into expression vectors pB7FWG2,0 vector [41] for C-terminal GFP-fusion and expression in plants; pDESTT17 vector (Invitrogen) for N-terminal fusion to a 6× his tag and inducible expression in bacteria.

Generation of coding sequences of AtMTM1 with mutations in the active site was carried out by a site directed mutagenesis approach using an overlap extension PCR approach [42]. The following primers overlapping by 42 bp in reverse orientation were designed to contain point mutations of residues of the conserved CX5R phosphatase domain active site:

(*PTP-fwd* 5′-CTTGTGCACAGCAGTGATGGATGGGTCAGAAC-3′ and

*PTP-rev* 5′-GTTCTGACCCATCCATCACAGCAGTGCACAAG-3′). In a first step the *rid-attB1* primer

5′-GGGGACAAGTTTGTACAAAAAAGCAGGCTTAATGACGCCGCCGAGACCACCG-3′ and the *PTP-rev* primer were used to obtain a 757 bp PCR product A.

The *PTP-fwd* primer 5′-CTTGTGCACAGCAGTGATGGATGGGTCAGAAC-3′ and a AtMTM-attB2 primer 5′-GGGGACCACTTTGTACAAGAAAGCTGGGTATTTAGGTTGGAAATAGCTATCG-3′ were used to amplify a 1228 bp PCR product B. The overlapping PCR products A and B were used as templates for a second step overlapping PCR, in the presence of primers *rid-attB1* and AtMTM*-attB2* to generate a 1953 bp gateway PCR product. After recombination cloning into the pDONR221 vector, several entry clones were sequenced and the two active site mutations of interest defined as mut1 and mut2 were selected for further studies.

### Phosphatase activity

Phosphoinositide 3-phosphatase assays were performed using the malachite green assay [43], [44] Clones obtained as described above were introduced into the pGEX4T-1 vector (GE Healthcare), expressed as GST-fusions in *E. coli* BL21 cells, and used to assay the enzyme activity. Recombinant affinity-column purified GST- tagged proteins were incubated with substrates (2.5 µmoles per experimental point) in 50 µl assay buffer (50 mM TrisHCl, pH 8.0, 100 mM KCl and 2 mM DTT). Di-C8 phosphoinositides (Echelon Biosciences Inc. catalogue numbers P-3008, P-5008, and P-3508) were used as substrates and PTEN lipid phosphatase (Echelon Biosciences Inc. catalogue number E-3000) was used as a positive control. Following incubation at 37°C for 30 minutes the reactions were quenched by the addition of 20 µl of 0.1 M *N*-ethylmaleimide and spun at 18,000 *g* for 10 minutes. Twenty-five microliters of the supernatant was added to 100 µl of malachite green reagent and vortexed. Samples were allowed to incubate for 40 minutes for color development before measuring absorbance at 620 nm. Inorganic phosphate release was measured by a standard curve of KH_2_PO_4_ in distilled water.

### Tobacco transient assays

Agrobacteria colonies containing binary plasmids for plant transformation were grown overnight in 10 mls of media with antibiotics. The cells were collected and re-suspended in an equal volume of induction medium (60 mM K_2_HPO_4_, 33 mM KH_2_PO_4_, (NH_4_)SO_4_, 1.7 mM Na Citrate.2H_2_O, 10 mM MES, 1 mM MgSO_4_, 0.2% Glucose, 0.5% Glycerol, antibiotics and 50 µg/ml of acetosyringone), and incubated with shaking for 6 hours at 30°C. The cells were re-suspended to an OD of 0.8 in infiltration medium (0.5× MS, 10 mM N-moropholino-ethanesulfonic acid, 150 uM acetosyringone) and used to infiltrate the abaxial surface of *N. benthamiana* leaves. After 40 hours detection of expression was conducted by laser scanning confocal microscopy using 488- and 633-nm excitation and two-channel measurement of emission, 522 nm (green/GFP) and 680 nm (red/chlorophyll). RFP was detected by excitation at 540 nm and emission at 590 nm.

### Real time quantitative RT-PCR analysis

RNA for qRT-PCR (quantitative real-time reverse transcription-polymerase chain reaction) was isolated with TRIZOL (Invitrogen) and purified with a RNeasy plant mini kit (Qiagen, catalogue number 74903). For the first strand cDNA synthesis 8 µg total RNA was treated with DNase I, extracted with phenol and chloroform, precipitated with ethanol, followed by the addition of oligo (dT) and superscript III reverse transcriptase (Invitrogen). Real-time PCR analysis was performed using the iCycleriQ real-time PCR instrument (Bio-Rad) and iQ SYBR Green Supermix (BioRad). The relative expressions of specific genes were quantitated using 2^−ΔΔCt^ calculation, where ΔCt is the difference in the threshold cycles of the test and housekeeping gene *ACTIN7*. ΔCt is the threshold cycle of the target gene subtracted from the threshold cycle of the housekeeping gene. The mean threshold cycle values for the genes of interest were calculated from three experiments. The following primers were used:

ACTIN7: F-(5′-CTACGAGGGGTATGCTCTTCCTCAT-3′), R- (5′- CTGAAGAACTGCTCTTGGCTGTCTC-3′) ATX1: F- (5′-CCCAATTGCTATTCTCGAGTCATCA-3′), R- (5′- TTTGCATGTTGTTCTTCAGCTTCTG-3′), ATMTM: F- (5′-CCCAAGGAGCTCTCTGGAGAATAAC-3′), R- (5′-CTTCCGACATGAGCACATCCTACTT-3′)

WRKY70: F- (5′-AGCAACTCCTCTCTCAACCCG-3′), R-(5′-CCATTGACGTAACTGGCCTGA-3′)

### Chromatin Immunoprecipitation (ChIP) assays

A protocol previously described [45] was used. Anti-H3K4me3 antibodies (Millipore, catalogue number 07–473) were used. Negative control samples were treated in the same way except that antibodies were not added. Each immunoprecipitation was performed in at least three separate experiments. Calibration curves were constructed to determine optimal amounts of chromatin to be used in each experiment and to ensure equivalent amounts of starting material. The PCR products were amplified using the primer sequences for

*WRKY70*: F (5′-agcaactcctctctcaacccg-3′); R: (5′-ccattgacgtaactggcctga-3′);

*ACTIN7*: F (5′-ggtgaggatattcagccacttgtctg-3′); R: (5′-tgtgagatcccgacccgcaagatc-3′).

### Mass assay; TLC; HPLC; PIP4K assay; 5′-phosphatase assay

Leaves were carefully excised from growing plants and immediately weighed. Total lipids were extracted from leaves either immediately or after dehydration stress (left on the bench for 2 hours at room temperature resulting in a weight loss of 30–40%) using ice-cold chloroform (250 µl) and methanol (500 µl) in a UCD-200TM bioruptor (Tosho Denki Co., Japan) operating at full power for 2 minutes. After storage at −20°C overnight another sonication step ensured complete leaf tissue disruption. Water (250 µl), 2.4 M HCl (200 µl) and CHCl_3_ (250 µl) were subsequently added to induce phase separation. After thorough mixing and centrifugation (5,000 *g* for 2 minutes at room temperature), the lower chloroform phase was washed once with theoretical upper phase and then dried in a speedvac at room temperature. PtdIns5*P* was quantitated in a neomycin affinity chromatography enriched phosphoinositide fraction using a radioenzymatic assay [6], [46]. The mass of PtdIns5*P* was normalized according to the starting leaf mass. Deacylated radiolabeled phospholipids were also analyzed by HPLC [17]. Purified yeast YNK-5 phosphatase was used to de-phosphorylate the isolated PtdIns*P*_2_ formed from the phosphorylation of endogenous leaf PtdIns*P* by PIP4Kα [17]. The TLC plate was developed once in chloroform/methanol/25% ammonia/water (ratio: 45/35/2/8, v/v/v/v). After air-drying for 30 minutes the plate was exposed to a phosphorimaging screen or to X ray film. HPLC analysis used a gradient of 0–1 M ammonium phosphate pH 3.85 with a flow rate of 1 ml/minute. Direct on-line radioactivity detection every 15 seconds avoided the requirement for fraction collecting. Correct peak identification was achieved using in-house radioactively labelled standards. The method has been described previously [46].

### Statistical analysis

A Wilcoxon signed t-rank test was applied for determining significant differences [23] employing significance threshold of P<0.05.

As biological material was taken from different plants, the measurements represented statistically relevant averages of PtdIns5*P* content in all the tested samples.
